# The role of DNAJC3 in enhancing glioma progression and regulating the tumor immune microenvironment

**DOI:** 10.1016/j.jbc.2025.111059

**Published:** 2025-12-13

**Authors:** Yinmin Shi, Wenxia Wang, Chenyang Liu, Niannian Wu, Xinyue Wei, Liang Wang, Huijuan Wang

**Affiliations:** 1 National Engineering Research Center for Miniaturized Detection Systems, College of Life Sciences, Northwest University, Xi’an, Shaanxi, China; 2 Department of Research and Development, Shaanxi Lifegen Co Ltd, Xi'an, China; 3 Key Laboratory of Resource Biology and Biotechnology in Western China, Ministry of Education, Northwest University, Xi’an, Shaanxi, China; 4 Department of Neurosurgery, Tangdu Hospital of Air Force Medical University, Xi’an, Shaanxi, China

**Keywords:** glioma, endoplasmic reticulum stress, DNAJC3, tumor immune microenvironment, proteomic analysis

## Abstract

Endoplasmic reticulum stress (ERS) is one of the important characteristics of tumors. Studies have demonstrated that ERS-related proteins play crucial roles in tumor initiation, progression, and immune infiltration. However, the specific role of ERS-induced DNAJC3 (DnaJ heat shock protein family [heat shock protein 40] member C3) in glioma remains unclear. In this study, we employed proteomic profiling combined with public databases to screen for differentially expressed proteins and found that DNAJC3 was significantly overexpressed in glioma. Subsequently, a series of cellular functional experiments were conducted to validate the important role of DNAJC3 in the malignant progression of glioma. In terms of mechanism, we found that DNAJC3 may exert its oncogenic effects by activating the mitogen-activated protein kinase–extracellular signal–regulated kinase and PI3K–AKT signaling pathways. Further in-depth analysis of the relationship between DNAJC3 expression levels and immune cell infiltration in glioma revealed that DNAJC3 may possess immunosuppressive properties. In addition, drug sensitivity analysis suggested that DNAJC3 expression may be associated with resistance to mitogen-activated protein kinase kinase inhibitors in glioma patients. In conclusion, this study found that DNAJC3 may play a significant role in the malignant progression and immune evasion of glioma, suggesting its potential as a therapeutic target. This finding is of great significance for understanding the role of DNAJC3 in the malignant progression of glioma and its oncogenic mechanisms. Moreover, it provides a theoretical basis for further exploring the immune evasion mechanisms of glioma and optimizing personalized treatment strategies.

Glioma is a primary tumor of the central nervous system originating from glial cells (1). The 2021 Fifth Edition of the World Health Organization (WHO) Classification of Tumors of the Central Nervous System categorizes gliomas into four grades (1, 2, 3, 4) by integrating histological features with molecular genetic markers (2, 3). Glioblastoma (GBM) is the most prevalent primary intracranial malignant tumor, characterized by aggressive growth, high mortality, and frequent recurrence (4). Currently, clinical management employs maximal safe resection combined with multimodal therapy (including radiotherapy and temozolomide chemotherapy) (5, 6). However, patient outcomes remain poor, with nearly 70% of patients experiencing tumor recurrence within 12 months postsurgery, a 5-year survival rate below 10%, and a median overall survival of only 12 to 15 months (7, 8). Therefore, identifying potential therapeutic targets has significant clinical significance for improving the prognosis of glioma patients and developing novel treatment strategies.

Endoplasmic reticulum stress (ERS) is an adaptive response by cells to cope with endoplasmic reticulum (ER) dysfunction or protein misfolding (9). In the tumor microenvironment, factors such as chronic hypoxia, oxidative stress, acidic conditions, and nutrient deprivation (*e.g.*, amino acid or glucose deficiency) collectively disrupt ER protein-folding homeostasis, leading to misfolded protein accumulation in the ER lumen (10). This triggers the unfolded protein response (UPR), which enhances protein-folding capacity and activates degradation pathways to restore ER proteostasis (11). DnaJ heat shock protein family (Hsp40) member C3 (DNAJC3) is an ER-resident cochaperone of binding immunoglobulin protein (BiP)–glucose-regulated protein 78 kDa (GRP78), also known as P58, P58IPK, or ERdj6 (12). Primarily localized within the ER lumen, DNAJC3 is induced during ER stress. Its N-terminal domain specifically recognizes and binds to the hydrophobic regions of misfolded proteins in the ER, whereas its C-terminal J-domain directly interacts with the ATPase domain of BiP–GRP78 to form a functional complex (13). As an ERS-associated protein, DNAJC3 dynamically maintains ER protein-folding homeostasis by mediating the synergistic action of these domains and participating in the molecular chaperone network of UPR. In neurodegenerative diseases, such as Alzheimer's disease and Parkinson's disease, DNAJC3 has been demonstrated to be involved in the regulation of Tau protein homeostasis (14). In diabetes, DNAJC3 is confirmed to participate in the protein-folding process and the maintenance of ER homeostasis in pancreatic β-cells. Dysfunction of DNAJC3 may exacerbate ERS in β-cells and promote β-cell apoptosis, thereby reducing insulin secretion and further aggravating disease progression (15). In addition, Tang *et al*. (16) have found that DNAJC3 can attenuate the apoptotic signaling pathways mediated by dsRNA and tumor necrosis factor-alpha, thereby promoting cell survival. This suggests that DNAJC3 may possess antiapoptotic capabilities, which could play a significant role in the malignant progression of tumors (16). In a study on osteosarcoma, Liang *et al*. (17) also discovered that the long noncoding RNA DNAJC3-AS1 can enhance the malignant phenotypes and chemotherapy resistance of osteosarcoma cells, thus promoting the progression of osteosarcoma. Notably, when DNAJC3 is knocked out, these oncogenic phenotypes are reversed, indicating that DNAJC3 may be a potential oncogenic driver (17). Recent evidence indicates that UPR signaling is hyperactivated in gliomas and is closely associated with tumor cell resistance to radiotherapy and chemotherapy (18, 19). However, the precise role of DNAJC3 in the progression of gliomas and the underlying mechanisms by which it exerts its effects currently remain unclear.

In this study, proteomic techniques and bioinformatics analysis were employed to confirm a significant overexpression of DNAJC3 in glioma tissues, which is associated with poor patient prognosis. Through a series of cellular functional experiments, we further investigated the regulatory role of DNAJC3 on the malignant phenotypes of gliomas, including proliferation, migration, invasion, and apoptosis, thereby elucidating its crucial role in the malignant progression of gliomas. In addition, bioinformatics analysis was utilized to analyze the impact of DNAJC3 expression on the immune microenvironment of gliomas. These findings not only enhance our understanding of the role of DNAJC3 in glioma progression and its underlying oncogenic mechanisms but also provide a theoretical basis for the development of novel therapeutic targets against gliomas.

## Results

### The ERS-related protein DNAJC3 is significantly overexpressed in glioma

**Figure 1:**
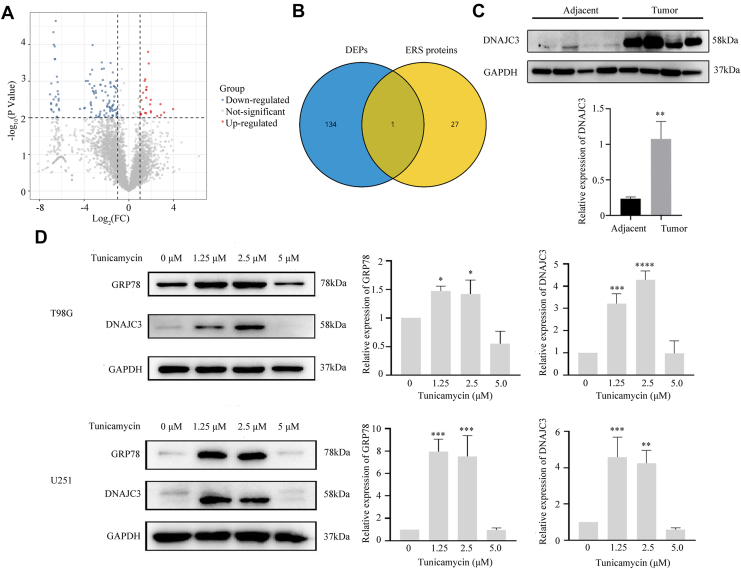
**The ERS-related protein DNAJC3 is significantly overexpressed in glioma.***A*, volcano map showing the differentially expressed proteins (|log_2_FC| ≥1, and *p* < 0.01). *B*, Venn diagram analysis of differentially expressed proteins and ERS-related proteins (relevance score >2). *C*, expression levels of DNAJC3 in glioma tissues (n = 3, Student's *t* test, compared with adjacent). *D*, changes in the expression levels of GRP78 and DNAJC3 in T98G and U251 cells after treatment with different concentrations of tunicamycin (n = 3, ANOVA, compared with 0 μM). The data are presented as mean ± SD. (∗∗∗∗*p* < 0.0001; ∗∗∗*p* < 0.001; ∗∗*p* < 0.01; ∗*p* < 0.05). DNAJC3, DnaJ heat shock protein family member C3; ERS, endoplasmic reticulum stress; FC, fold change.

We conducted label-free quantitative proteomic analysis on three cases of primary GBM tissues and three cases of adjacent nontumor tissues using LC–MS/MS. A total of 4456 proteins were identified (Fig. 1*A*). Among them, compared with adjacent nontumor tissues, 25 proteins were significantly upregulated in GBM tissues (Fig. S2*A*), whereas 110 proteins were significantly downregulated (|log_2_ fold change| ≥1 and *p* < 0.01) (Fig. S2*B*). To investigate the relationship between ERS and the malignant progression of glioma, we downloaded 1678 ERS-related proteins from the GeneCards database (https://www.genecards.org/) (Table S1). Of these ERS-related proteins, there were 28 proteins with a relevance score >2 (Fig. S1*C*). Venn diagram analysis further identified DNAJC3 as a key differentially expressed protein (Fig. 1*B*). Western blotting (WB) was used to verify the expression of DNAJC3 in GBM tissues, which also showed significant overexpression of DNAJC3 (Fig. 1*C*). Subsequently, by integrating the quantitative information of immunohistochemical images from the Human Protein Atlas public database, the expression levels of DNAJC3 in normal tissues and glioma tissues were further verified. The results showed that the expression level of DNAJC3 in glioma tissues was significantly higher than that in normal brain tissues, which was consistent with the previous experimental results (Fig. S2). In addition, an ERS *in vitro* model was established by treating GBM cells with tunicamycin. Subsequently, WB was employed to determine the expression of the ERS marker protein BiP–GRP78 and the differentially expressed protein DNAJC3. The results revealed that the expression levels of both BiP–GRP78 and DNAJC3 initially increased and then decreased after 24 h of treatment with different concentrations of tunicamycin (Fig. 1*D*). Furthermore, treatment of GBM cells with a low concentration of tunicamycin (2.5 μM) resulted in a time-dependent and sustained upregulation of the expression of the ERS markers BiP–GRP78 and DNAJC3. In contrast, a higher concentration of tunicamycin (5 μM) induced a biphasic expression pattern for these proteins, characterized by an initial significant increase followed by a subsequent decline (Fig. S1, *D* and *E*). This reduction is highly likely attributable to the decompensation of the cellular stress response, leading to the induction of programmed cell death (apoptosis). Collectively, these findings suggest that the ER stress–induced aberrant expression of DNAJC3 may play a pivotal regulatory role in the malignant progression of glioma.

### A high level of DNAJC3 expression is associated with poor prognosis in glioma patients

**Figure 2:**
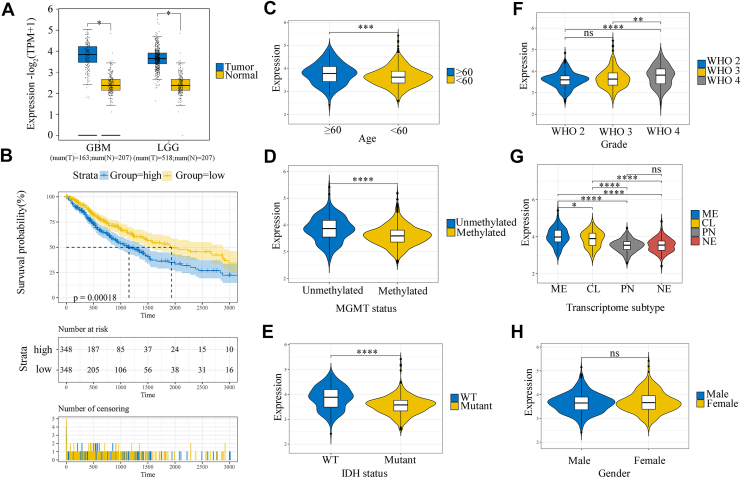
**DNAJC3 was associated with clinicopathological characteristics of glioma patients in the TCGA database.***A*, expression levels of DNAJC3 in glioma tissues and normal brain tissues in the GEPIA2 database (Student's *t* test). *B*, the relationship between high/low expression of DNAJC3 and the survival status of glioma patients (Log-rank test). *C*–*H*, the relationship between high/low expression of DNAJC3 and the clinicopathological characteristics of glioma patients, including age (*C*), MGMT promoter methylation status (*D*), IDH mutation status (*E*), WHO grade (*F*), transcriptome subtype (*G*), and gender (*H*) (Student's *t* test/ANOVA). (∗∗∗∗*p* < 0.0001; ∗∗∗*p* < 0.001; ∗∗*p* < 0.01; ∗*p* < 0.05; ns, no significance). DNAJC3, DnaJ heat shock protein family member C3; IDH, isocitrate dehydrogenase; MGMT, methylguanine-DNA methyltransferase; TCGA, The Cancer Genome Atlas; WHO, World Health Organization.

We further analyzed the expression levels of DNAJC3 in glioma tissues and normal brain tissues using the GEPIA2 database (http://gepia2.cancer-pku.cn/). The results also demonstrated a significant increase in DNAJC3 in glioma tissues (Fig. 2*A*). Based on the transcriptome sequencing data of 702 glioma patients from The Cancer Genome Atlas (TCGA) database, we explored the correlation between DNAJC3 and the clinicopathological characteristics of glioma patients. Kaplan–Meier survival analysis revealed that the overall survival rate of the high-DNAJC3 expression group was significantly lower than that of the low-DNAJC3 expression group (*p* = 0.00018) (Fig. 2*B*). Clinicopathological correlation analysis showed that, except for gender (Fig. 2*H*), the expression level of DNAJC3 was significantly correlated with age (Fig. 2*C*), methylguanine-DNA methyltransferase promoter methylation status (Fig. 2*D*), isocitrate dehydrogenase (IDH) mutation status (Fig. 2*E*), WHO grade (Fig. 2*F*), and transcriptome subtype (Fig. 2*G*) of glioma patients. Subsequently, we used the transcriptome sequencing data of 1018 glioma patients from the Chinese Glioma Genome Atlas (CGGA) database as a validation set. The results also confirmed a significant correlation between DNAJC3 expression and the clinicopathological characteristics of glioma patients, which was consistent with the findings based on the TCGA database (Fig. S3).

### DNAJC3 promotes the malignant progression of glioma

**Figure 3:**
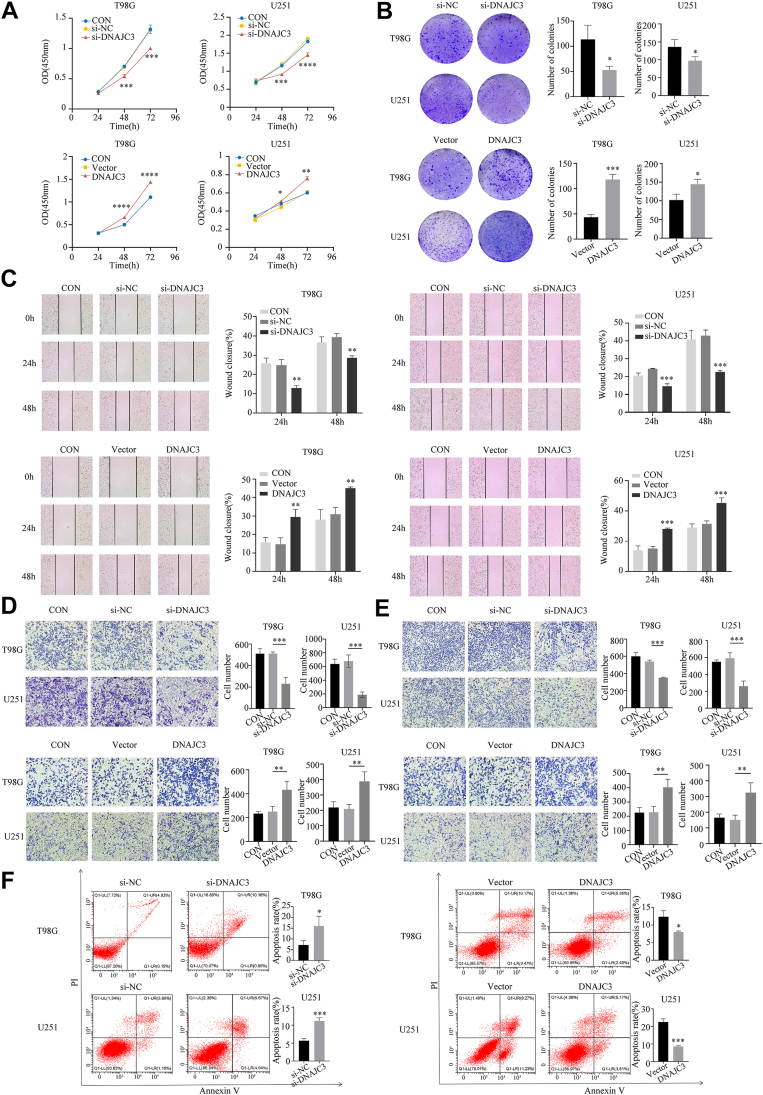
***In vitro*, overexpression of DNAJC3 promotes the proliferation, migration, invasion, and antiapoptosis ability of T98G and U251 cells.***A*, effects of high/low expression of DNAJC3 on cell viability were assessed using the CCK-8 assay (n = 3, Student's *t* test, compared with si-NC/vector). *B*, the impact of high/low expression of DNAJC3 on cell proliferation capacity was examined through colony formation assay (n = 3, Student's *t* test, compared with si-NC/vector). *C*, the influence of high/low expression of DNAJC3 on cell migration ability was evaluated using a wound healing assay (n = 3, ANOVA, compared with si-NC/vector). *D*, the effect of high/low expression of DNAJC3 on cell migration was assessed using a transwell assay (magnification: 40×) (n = 3, ANOVA, compared with si-NC/vector). *E*, the impact of high/low expression of DNAJC3 on cell invasion ability was determined using a transwell invasion assay (n = 3, ANOVA, compared with si-NC/vector). *F*, the effect of high/low expression of DNAJC3 on cell apoptosis was analyzed using flow cytometry (n = 3, Student's *t* test, compared with si-NC/vector). The data are presented as mean ± SD. (∗∗∗∗*p* < 0.0001; ∗∗∗*p* < 0.001; ∗∗*p* < 0.01; ∗*p* < 0.05; CON, control group; DNAJC3, DNAJC3 overexpression group; si-DNAJC3, DNAJC3 knockdown group; si-NC, negative control of the knockdown group; vector, empty vector control of the overexpression group.) CCK-8, Cell Counting Kit-8; DNAJC3, DnaJ heat shock protein family member C3.

To investigate the regulatory role of DNAJC3 in the malignant phenotypes of glioma, we successfully constructed *DNAJC3* knockdown and overexpression cell models (Fig. S4). We evaluated the cell proliferation ability using Cell Counting Kit-8 (CCK-8) and colony formation assays, assessed the cell migration ability through wound healing and transwell migration assays, examined the changes in cell invasion ability *via* transwell invasion assays, and determined the effect of DNAJC3 on the apoptosis ability of GBM cells using flow cytometry. The results revealed that overexpression of DNAJC3 significantly enhanced the proliferation, migration, invasion, and antiapoptosis abilities of GBM cells. In contrast, the knockdown of DNAJC3 reversed these effects (Fig. 3). These findings suggest that DNAJC3 may play a crucial role in the malignant progression of glioma.

### DNAJC3 promotes the survival of glioma cells by activating the mitogen-activated protein kinase–extracellular signal–regulated kinase and PI3K–AKT signaling pathways

**Figure 4:**
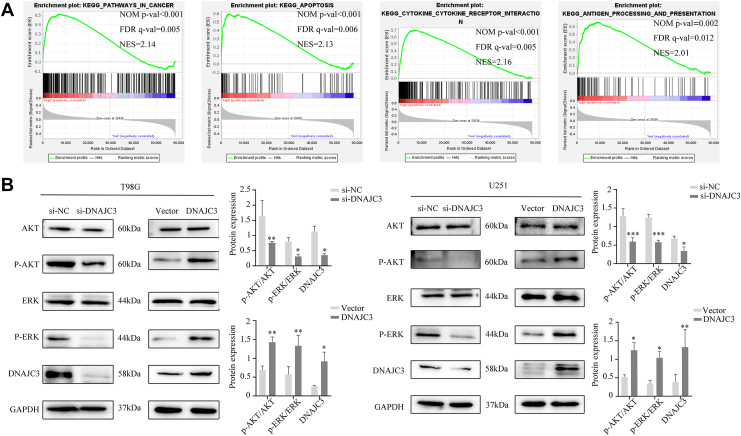
**DNAJC3 promotes the survival of glioma cells by activating the MAPK–ERK and PI3K–AKT signaling pathways.***A*, enrichment analysis of GSEA. *B*, WB was used to detect the effects of silencing or overexpression of DNAJC3 on the phosphorylation levels of ERK and AKT (n = 3, Student's *t* test, compared with si-NC/vector). The data are presented as mean ± SD. (∗∗∗*p* < 0.001; ∗∗*p* < 0.01; ∗*p* < 0.05; DNAJC3, DNAJC3 overexpression group; si-DNAJC3, DNAJC3 knockdown group; si-NC, negative control of the knockdown group; vector, empty vector control of the overexpression group.) DNAJC3, DnaJ heat shock protein family member C3; ERK, extracellular signal–regulated kinase; GSEA, gene set enrichment analysis; MAPK, mitogen-activated protein kinase; WB, Western blot.

We utilized gene set enrichment analysis (GSEA) to explore the molecular mechanism by which DNAJC3 regulates the malignant progression of glioma. The results revealed that the DNAJC3 high-expression group was primarily enriched in multiple tumor–related and immune-related pathways (Table S2). Subsequently, the Kyoto Encyclopedia of Genes and Genomes pathway analysis indicated that the core regulatory nodes of tumor-related pathways were concentrated in the mitogen-activated protein kinase (MAPK)/extracellular signal–regulated kinase (ERK) and PI3K–AKT signaling pathways (Fig. 4*A*). This implies that DNAJC3 may drive the progression of glioma by activating these two classical oncogenic signaling pathways. We further examined the changes in the phosphorylation levels of ERK and AKT using WB. The results showed that knocking down DNAJC3 significantly inhibited the phosphorylation levels of ERK and AKT, whereas overexpressing DNAJC3 significantly enhanced their phosphorylation levels (Fig. 4*B*). This suggests that DNAJC3 may promote the malignant progression of glioma by activating the MAPK–ERK and PI3K–AKT signaling pathways.

### The high expression of DNAJC3 promotes the formation of an immunosuppressive tumor microenvironment in glioma

**Figure 5:**
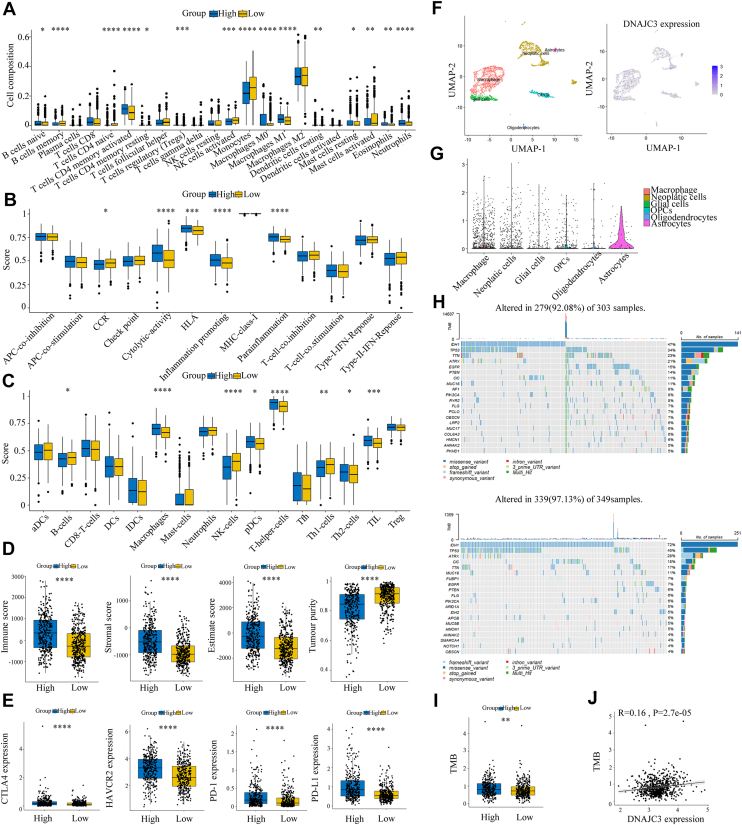
**The high expression of DNAJC3 is associated with the formation of an immunosuppressive tumor microenvironment and a higher TMB in glioma.***A*, boxplots showing the expression levels of 22 immune cell subsets in the high and low DNAJC3 expression groups. *B* and *C*, boxplots of immune-related functions or pathways (*B*) and immune cell infiltration (*C*) in high and low DNAJC3 expression groups. *D*, analysis of immune score, stromal score, estimate score, and tumor purity in glioma patients using the ESTIMATE algorithm. *E*, the relationship between DNAJC3 expression levels and four immune checkpoints. *F*, identification of cell types through single-cell sequencing analysis. *G*, differences in DNAJC3 expression levels in different cell clusters. *H*, tumor mutation waterfall plots for high and low DNAJC3 expression groups. *I*, the relationship between high and low DNAJC3 expression groups and TMB. *J*, correlation analysis between DNAJC3 expression and TMB (Pearson's correlation analysis). (Student's *t* test, ∗∗∗∗*p* < 0.0001; ∗∗∗*p* < 0.001; ∗∗*p* < 0.01; ∗*p* < 0.05.) DNAJC3, DnaJ heat shock protein family member C3; TMB, tumor mutation burden.

Given that multiple immune-related signaling pathways were significantly associated with the DNAJC3 high-expression group (Fig. 4*A*), we further explored the relationship between DNAJC3 and the glioma immune microenvironment using various algorithms. The Cibersort algorithm revealed that in the DNAJC3 high-expression group, there was less infiltration of immune cells with antitumor effects, such as B cells, T cells, NK cells, and type 1 helper T cells. In contrast, there was an increased infiltration of immune cells with immunosuppressive functions, such as regulatory T cells (Treg) and type 2 helper T cells (Fig. 5*A*). This result suggests that high expression of DNAJC3 may have an immunosuppressive effect. Single-sample GSEA analysis showed that in the DNAJC3 high-expression group, some biological processes related to proinflammation and protumor were more active, such as inflammation promoting and parainflammation (Fig. 5, *B* and *C*). The Estimate score indicated that the DNAJC3 high-expression group had higher immune scores, stromal scores, and lower tumor purity (Fig. 5*D*). In addition, the expression of four common immune checkpoints (PD-L1, CTLA4, HAVCR2, and PD-1) was significantly upregulated in the DNAJC3 high-expression group (Fig. 5*E*). The aforementioned results suggest that the immune function of patients in the DNAJC3 high-expression group may be suppressed, indicating that DNAJC3 may play an important role in glioma immune evasion.

We further analyzed the distribution of DNAJC3 in the glioma immune microenvironment based on single-cell sequencing data. The results showed that DNAJC3 was specifically highly expressed mainly in macrophages and astrocytes (Fig. 5, *F* and *G*), suggesting that these two types of cells may be the key effector cells through which DNAJC3 regulates the glioma immune microenvironment.

### The high expression of DNAJC3 is associated with higher tumor mutation burden

In glioma, tumor mutation burden (TMB) often exhibits a negative correlation with patient prognosis (20). Therefore, we compared the gene mutation profiles and TMB between the DNAJC3 high-expression and low-expression groups. The results showed that in the DNAJC3 high-expression group, frequently mutated genes included IDH1 (47%), TP53 (34%), TTN (23%), ATRX (21%), and EGFR (15%), whereas the low-expression group was mainly characterized by mutations in IDH1 (72%), TP53 (45%), ATRX (28%), CIC (18%), and TTN (17%) (Fig. 5*H*). Notably, the TMB in the DNAJC3 high-expression group was significantly higher than that in the low-expression group (Fig. 5*I*), and there was a positive correlation between the expression level of DNAJC3 and TMB (Pearson's *r* = 0.16, *p* < 0.05) (Fig. 5*J*).

### Drug sensitivity analysis of DNAJC3

**Figure 6:**
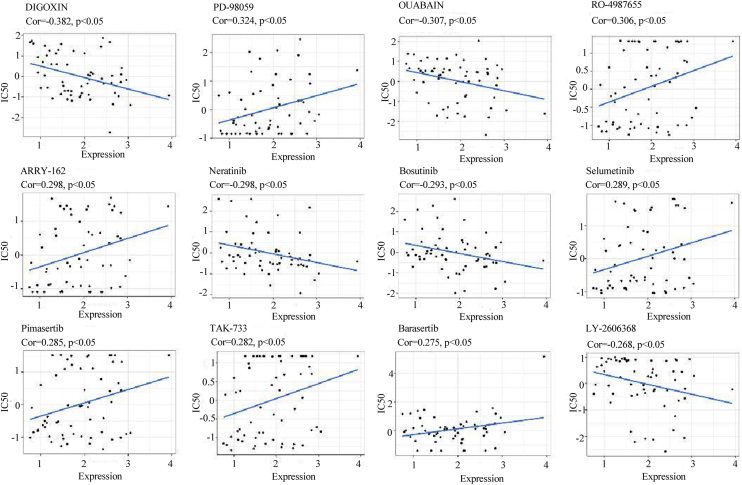
**Drug sensitivity analysis of DNAJC3.** The scatter plots illustrate the relationship between the sensitivity of glioma patients to different drugs and the expression levels of DNAJC3. (Pearson's correlation analysis; Cor, correlation coefficient.) DNAJC3, DnaJ heat shock protein family member C3.

Typically, a higher TMB indicates the presence of more genetic mutations within tumor cells, which may lead to increased sensitivity of tumor cells to some molecular targeted drugs. Based on the CellMiner database, we investigated the impact of DNAJC3 on drug sensitivity in tumor patients. The results showed that high expression of DNAJC3 could enhance the sensitivity of tumor cells to cardiac glycosides (such as digoxin and ouabain) and kinase inhibitors (such as neratinib and bosutinib), while reducing the sensitivity of tumor cells to MEK inhibitors (such as PD-98059, RO-4987655, ARRY-162, selumetinib, pimasertib, and TAK-733) (Fig. 6). This finding may provide new insights for individualized drug therapy in glioma patients.

## Discussion

ERS, one of the important characteristics of tumors, is closely associated with the malignant progression and immune infiltration of tumors (21). Studies have shown that the abnormal activation of UPR can accelerate tumor progression by enhancing the proliferation, migration, and invasion of glioma cells (22, 23). As an ERS-related protein, previous studies on DNAJC3 have mainly focused on its protective role in maintaining protein homeostasis by regulating eukaryotic Initiation Factor 2 alpha dephosphorylation in metabolic diseases (24, 25). However, its specific role in the progression of glioma remains unclear.

In this study, which utilized LC–MS/MS technology, we found that the ERS-related protein DNAJC3 was significantly overexpressed in glioma tissues. Analysis of the glioma sequencing data from the TCGA database revealed a marked association between high expression of DNAJC3 and poor prognosis in glioma patients, suggesting that DNAJC3 might be a potential oncogenic factor. Similarly, Wang *et al.* (26), when constructing a prognostic model for apoptosis-related genes in oral squamous cell carcinoma, identified six risk biomarkers associated with oral squamous cell carcinoma prognosis, with DNAJC3 being one of them, which is consistent with our analysis results (26). Further research demonstrated that overexpression of DNAJC3 significantly enhanced the proliferation, migration, invasion, and antiapoptotic abilities of GBM cells, whereas the depletion of DNAJC3 yielded the opposite outcomes. In addition, multiple studies have confirmed that long noncoding RNA DNAJC3-AS1 could promote the progression of various solid tumors, including breast cancer, hepatocellular carcinoma, colorectal cancer, renal cell carcinoma, and thyroid cancer, by regulating the expression of DNAJC3 (27, 28, 29, 30, 31). Collectively, these findings further indicate that DNAJC3 possesses oncogenic properties.

The results of GSEA indicated significant enrichment of multiple tumor–related and immune-related signaling pathways in the DNAJC3 high-expression group. In an analysis of ERS-related signatures associated with clinical prognosis and immunotherapy in glioma, Li *et al.* (32) similarly confirmed that ERS-related gene signatures are closely associated with the malignant progression and immune infiltration of gliomas (32). WB further validated that the MAPK–ERK and PI3K–AKT signaling pathways are involved in the process of DNAJC3-driven malignant progression of gliomas. Notably, in the context of human epidermal growth factor receptor 2 (HER2)–positive breast cancer patients' response to anti-HER2 therapy, Ji *et al*. (33) also discovered that DNAJC3 can regulate the HER2 response kinase pathway through the PI3K and MAPK signaling pathways. Although our data strongly suggest that DNAJC3 affects cell phenotype by regulating the MAPK–ERK and PI3K–AKT signaling pathways, we currently cannot completely rule out the potential impact of DNAJC3 on the overall state of the ER.

As is widely known, glioma is a typical "cold" tumor in terms of immunity, with few lymphocyte infiltrations in its immune microenvironment but a large number of immune-suppressive components (34). This leads to an immunosuppressive state in the tumor microenvironment, which is also the reason why most glioma patients are insensitive to immunotherapy (35). In our study, we found that high expression of DNAJC3 is closely associated with the infiltration of immune cells in glioma. Specifically, there is a decrease in the infiltration of lymphocytes with antitumor activity, whereas an increase in the infiltration of tumor-infiltrating immune cells with immunosuppressive functions. This suggests that high expression of DNAJC3 may promote the formation of an immunosuppressive tumor microenvironment in glioma. Further analysis revealed that processes conducive to tumor immune evasion are more active in the high DNAJC3 expression group, and tumor purity is also lower. Previous studies have shown that lower tumor purity can further exacerbate immunosuppression (36), which may explain why the efficacy of immunotherapy in glioma patients is often unsatisfactory. In addition, the expression of immune checkpoint molecules is significantly upregulated in patients with high DNAJC3 expression, which once again indicates that DNAJC3 expression is beneficial for the formation of an immunosuppressive tumor microenvironment. In fact, the relationship between ERS-related proteins and tumor immunosuppression has been reported previously. For example, Jiang *et al.* found that the ERS response protein TMED4 can maintain the stability of Foxp3 and the immunosuppressive function of Treg cells by mediating the levels of reactive oxygen species dependent on the IRE1α–XBP1 signaling axis. When TMED4 is depleted, Treg cells promote the activation of effector T cells in mouse tumor models, thereby enhancing the body's antitumor immunity (37). In GBM, Alessandra De Leo *et al*. (38) also discovered that the protein kinase R–like ER kinase can promote the immunosuppressive activity of monocyte-derived macrophages by facilitating glycolysis and regulating histone lactylation. In conclusion, the aforementioned results suggest that DNAJC3 may have an immunosuppressive role in the glioma immune microenvironment, thereby promoting immune evasion and malignant progression of glioma. This finding implies that DNAJC3 could be a potential therapeutic target, which is of great significance for the transformation of “cold” tumors into “hot” tumors. Meanwhile, it also provides a theoretical basis for our in-depth understanding of the immune evasion mechanisms in glioma.

TMB holds importance in evaluating the malignancy, growth rate, and treatment sensitivity of tumors. Generally, a low TMB often indicates that the tumor develops and progresses slowly, with relatively weak invasiveness and metastatic ability, a slower disease progression, and a relatively better prognosis for patients (20). We found that patients with high DNAJC3 expression had significantly higher TMB compared with those with low DNAJC3 expression, suggesting that patients with high DNAJC3 expression may have a poorer prognosis. This result is mutually corroborated with the previous Kaplan–Meier survival analysis findings. In the drug sensitivity analysis, we observed that high DNAJC3 expression markedly reduced the sensitivity of tumor cells to MEK inhibitors. As mentioned earlier, DNAJC3 could activate the MAPK–ERK signaling pathway. Therefore, we speculate that DNAJC3 may mediate the resistance of tumor cells to MEK inhibitors by activating the MAPK–ERK signaling pathway. This discovery may provide new insights for individualized medication strategies in glioma patients.

The main limitation of this study lies in the lack of validation through *in vivo* animal models. In addition, the current results only establish the correlation between DNAJC3 and the activation of the ERK–AKT pathway, whereas the specific molecular mechanism still requires further exploration. Moreover, the specific mechanism of how DNAJC3 regulates the immune microenvironment of glioma remains a key area for future research.

In summary, this study suggests that DNAJC3 may be a potential oncogene, and its high expression is associated with poor prognosis in glioma patients. Preliminary exploration has revealed that DNAJC3 may promote the malignant progression of gliomas by activating the MAPK–ERK and PI3K–AKT signaling pathways, as well as regulating immune cell infiltration. In addition, this study has identified a possible mechanism underlying MEK inhibitor resistance in patients with high DNAJC3 expression. These findings not only elucidate the crucial role of DNAJC3 in the progression of gliomas but also provide new insights for individualized medication strategies in glioma patients.

## Experimental procedures

### Clinical samples and cell lines

GBM tissues and adjacent nontumor tissues were obtained from Tangdu Hospital, Air Force Medical University. All tissues were rapidly frozen in liquid nitrogen and stored at −80 °C. This study was conducted with informed consent obtained from all participants, adhering to the relevant principles of the Declaration of Helsinki. The protocol was approved by the Ethics Committee of Tangdu Hospital.

The human GBM cell lines T98G and U251 were purchased from Procell Life Science & Technology Co, Ltd. The authenticity of these two cell lines was verified through short tandem repeat analysis. In addition, these cells were tested and confirmed to be free from mycoplasma contamination. T98G cells were cultured in MEM (containing NEAA) medium (Pricella) supplemented with 10% fetal bovine serum (Gibco), and U251 cells were cultured in DMEM high-glucose medium (Pricella), also supplemented with 10% fetal bovine serum. All cells were cultured at 37 °C and 5% CO_2_. Cells in the logarithmic growth phase were utilized for experimental procedures.

### Liquid chromatography–tandem mass spectrometry

After denaturation, reduction, and alkylation of the tissues, sequence-grade trypsin was used for digestion. Following centrifugation, the supernatant was collected, and peptide separation was performed using the Easy-nLCTM 1200 system (Thermo Fisher Scientific). Mass spectrometry analysis was conducted using the Orbitrap Fusion Lumos mass spectrometer (Thermo Fisher Scientific). The Sequest algorithm in Proteome Discoverer 2.3 software (Thermo Fisher Scientific) was utilized to search the mass spectrometry data in the UniProt database (http://www.uniprot.org). The search parameters were set as follows: the enzyme specificity was trypsin, which allowed two errors to be missed, and the false discovery rate for protein identification was set at 1%. The criteria for screening differentially expressed proteins were |log_2_ fold change| ≥1, and a *p* value <0.05 was considered statistically significant. The complete experimental procedures for the proteomic analysis and the corresponding identification results have been documented in Table S3, with the analytical workflow strictly adhering to the methods described in our previous study (39).

### Clinical correlation analysis

Based on the transcriptome sequencing data of 702 glioma samples from the TCGA database, we analyzed the correlation between DNAJC3 and clinical characteristics, including survival analysis, gender, age, transcriptional subtype, methylguanine-DNA methyltransferase promoter methylation status, IDH mutation status, and WHO classification. In addition, two sets of glioma transcriptome sequencing data, mRNAseq_693 and mRNAseq_325, were downloaded from the CGGA database. After removing batch effects using the R package "sva," a total of 1018 cases of glioma transcription sequencing data were collected and used as a validation dataset. Finally, the Human Protein Atlas database (http://www.proteinatlas.org/) was also used to confirm the local expression pattern of DNAJC3 in tissue samples.

### Quantitative PCR

Total RNA was extracted from glioma cells using an RNA extraction kit (Invitrogen, Thermo Fisher Scientific). The extracted RNA was then reverse-transcribed into complementary DNA using the TransScript All-in-One First-Strand Complementary DNA Synthesis SuperMix for qPCR (AT341; TRANS). qPCRs were performed using the PerfectStart Green qPCR SuperMix (AQ601; TRANS). The expression level of the DNAJC3 gene was analyzed using the 2^−ΔΔCt^ method. The primers were synthesized by Beijing Tsingke Biotechnology Co, Ltd, and the sequences were designed as follows. For DNAJC3, the forward primer was 5′-ACCTTTCCTCCTCTTCACTCG-3′, and the reverse primer was 5′-TCCACATTCAGCACCTTCGTA-3′. For GAPDH, the forward primer was 5′-GAGTCAACGGATTTGGTCGT-3′, and the reverse primer was 5′-GACAAGCTTCCCGTTCTCAG-3′.

### Western blotting

After washing the cells with prechilled PBS, the cells were lysed using radioimmunoprecipitation assay lysis buffer (Solarbio) to extract cellular proteins. The protein concentration was then quantified using a Bicinchoninic Acid Protein Assay Kit (Beyotime). The denatured proteins were subjected to SDS-PAGE. Following electrophoresis, the proteins were transferred onto a polyvinylidene fluoride membrane (Millipore). After blocking the membrane for 1 h in Tris-buffered saline with Tween-20 containing 5% nonfat milk, the membrane was incubated overnight at 4 °C with primary antibodies specific for DNAJC3 (1:1000 dilution, 26721-1-AP; Proteintech), GAPDH (1:2000 dilution, 60004-1-Ig; Proteintech), GRP78 (1:2000 dilution, 66574-1-Ig; Proteintech), AKT (1:1000 dilution, 10176-2-AP; Proteintech), p-AKT (1:1000 dilution, 66444-1-Ig; Proteintech), ERK (1:1000 dilution, 67170-1-Ig; Proteintech), and p-ERK (1:1000 dilution, 28733-1-AP; Proteintech). Subsequently, the membrane was washed and incubated with anti-rabbit or anti-mouse secondary antibodies (1:2000 dilution, SA00001-1/2; Proteintech) for 1 h at room temperature. Thereafter, an electrochemiluminescence kit (Bio-Rad) was used to detect the protein bands on an imaging system (Tanon-4600). The intensity of the protein bands was calculated using ImageJ (National Institutes of Health). The specificity of all antibodies was verified by the detection of a single band at the expected molecular weight. All experiments were independently repeated at least three times, and data are presented as the mean ± SD.

### Synthesis and transfection of plasmids and siRNA

siRNAs were purchased from Shanghai Genechem Co, Ltd to suppress the expression of the DNAJC3 gene, with the following sequences: DNAJC3 sense strand (DNAJC3-F): 5′-GUCGCAGAAACGAGAUUAUTT-3′ and DNAJC3 antisense strand (DNAJC3-R): 5′-AUAAUCUCGUUUCUGCGACTT-3′. The Puro-based recombinant plasmid containing the DNAJC3 sequence (plenti-UCOE-SFFV-zsgreen-P2A-Puro-DNAJC3) and a negative control plasmid were purchased from Xi'an Genecarer Biotechnology Co, Ltd for overexpression of DNAJC3 gene expression. Glioma cells were seeded in 6-well plates at a density of 2 × 10^5^ cells per well. When the cell density reached 70% to 80%, Lipofectamine 2000 was used to transfect the siRNAs or plasmids into the glioma cells at a concentration of 50 nM.

### Cell proliferation assay

Cell viability was assessed using the CCK-8 assay (TargetMol). After 48 h of transfection, the cells were digested and seeded into 96-well plates at a density of 2 × 10^3^ cells per well. Subsequently, the cell culture medium was replaced with 100 μl of serum-free medium containing 10 μl of CCK-8 solution at 0 h, 24 h, and 48 h, respectively. Following incubation at 37 °C for 1 h, the absorbance values were recorded at 450 nm using a microplate reader (Tecan Spark).

Cell proliferation capacity was assessed using a colony formation assay. Cells were seeded into 6-well plates at a density of 200 cells/ml and cultured in an incubator at 37 °C with 5% CO_2_. Once visible colonies had formed, the cells were fixed with 4% paraformaldehyde and stained with 0.1% crystal violet. The number of cell colonies was then counted using ImageJ.

### Wound healing assay

The lateral migration capacity of cells was evaluated using a wound healing assay. After 24 h of transfection, the cells were digested and seeded into 6-well plates. Once the cells had adhered to the plate, vertical lines were drawn across the cells. After aspirating the old medium, PBS was used to rinse and remove the dislodged cells. Subsequently, the complete medium was replaced with a serum-free medium. Images of the wound healing were captured under a microscope at 0 h, 24 h, and 48 h, respectively.

### Transwell cell migration and invasion assay

For the migration assay, cells were digested and resuspended in 200 μl of serum-free medium at a density of 5 × 10^5^ cells/ml, then seeded into the upper chamber (Corning Costar). The complete medium (500 μl) was added to the lower chamber. After 48 h of incubation in the incubator, the chamber was fixed with 4% paraformaldehyde for 30 min, stained with 0.1% crystal violet, and imaged under a microscope for cell counting. For the assessment of cell invasion ability, the procedure was the same as above, except that the upper chamber was precoated with Matrigel.

### Cell apoptosis assay

After digestion and cell collection, the single-cell suspension was prepared using a binding buffer. The procedure was carried out according to the instructions of the Annexin-FITC/propidium iodide apoptosis detection kit (F6012L; US Everbright Inc.), and the apoptosis status of the cells was detected using a flow cytometer (EPICS XL; Beckman Coulter, Inc).

### Gene set enrichment analysis

After downloading the RNA-Seq data of gliomas from the TCGA database, the samples were divided into two groups based on the expression level of DNAJC3. GSEA was then performed using GSEA 4.3.2 software (Broad Institute). The screening conditions were |normalized enrichment score| >1, the false discovery rate <0.25, and *p* value <0.05.

### Immune landscape analysis

Based on the RNA-Seq data of gliomas in the TCGA database, four immunoreaction algorithms were used to analyze the immune microenvironment of gliomas between the high- and low-expression DNAJC3 groups. The Cibersort algorithm was used to evaluate the proportions of 22 types of immune cells in different groups (40). Immune cell activity or immune function and immune pathway were calculated using single-sample GSEA. These marker genes were obtained from previous studies and are listed in Table S4 (41). The Estimate algorithm was used to calculate immune score, stromal score, estimation score, and tumor purity (42). Regarding the immune checkpoint, we analyzed the expression of four common immunosuppressants (PD-L1, CTLA4, HAVCR2, and PD-1) in different groups.

### Single-cell sequencing analysis

The single-cell sequencing data were obtained from the Gene Expression Omnibus (accession number: GSE84465), which performed single-cell RNA-Seq on 3589 cells in a cohort of four glioma patients (43). We processed the single-cell data expression matrix using the R package Seurat. After data normalization by NormalizeData, a total of 2000 highly variable genes were identified *via* the FindVariableGenes function. Subsequently, principal component analysis was performed using RunPCA, followed by FindNeighbors and FindClusters to cluster cells. The results were displayed using the Uniform Manifold Approximation and Projection method, and cellular annotation was performed based on highly variable genes in cell populations. Finally, the expression levels of DNAJC3 in different cell populations were visualized using VlnPlot and Dimplot (44).

### Gene mutation analysis

Based on the somatic mutation data of glioma samples from the TCGA database, we performed gene mutation analysis *via* the Maftools package (45). By calculating the TMB for each sample, we compared the differences in TMB between the high- and low-expression groups of DNAJC3. In addition, we analyzed the correlation between DNAJC3 gene expression and TMB.

### Drug sensitivity analysis

CellMiner is a web-based database resource for integrating diverse molecular data types and related metadata of NCI-60 (60 diverse human cancer cell lines) (46). The NCI-60 cell line is currently the most frequently utilized cancer cell sample group for testing anticancer drugs. In this study, we downloaded the drug sensitivity data and RNA-Seq data of the NCI-60 cell lines. Then, we employed Pearson's correlation analysis and Student's *t* test to explore the association between the expression of DNAJC3 and the sensitivity to common antitumor drugs.

### Statistical analysis

All statistical analyses were performed using R software (The R Foundation for Statistical Computing, version 4.2.2) and GraphPad Prism (GraphPad Software, version 8.0.1). The log-rank test was used in the Kaplan–Meier survival analysis. The correlation coefficients were calculated by Pearson's correlation analysis. Differences in quantitative data between the two groups were compared using *t* test. For comparisons among more than two groups, one-way ANOVA was used. *p* < 0.05 were considered statistically significant. All the experiments were replicated biologically three times. All results are presented as means ± SD.

## Data availability

Proteomics raw data have been uploaded to the ProteomeXchange Consortium (accession number: PXD026503). In addition, this study analyzed publicly available datasets, which can be found in the TCGA, CGGA, Gene Expression Omnibus datasets (accession number: GSE84465).

## Supporting information

This article contains supporting information.

## Conflict of interest

The authors declare that they have no conflicts of interest with the contents of this article.
