# Supplementary material for: The role of DNAJC3 in enhancing glioma progression and regulating the tumor immune microenvironment
Source: J Biol Chem. 2025 Dec 13;302(2):111059. doi: 10.1016/j.jbc.2025.111059 (PMC12805106; doi:10.1016/j.jbc.2025.111059)
Supplement: Supporting information [file mmc1.docx]

**The role of DNAJC3 in enhancing glioma progression and regulating the tumor immune microenvironment**

**
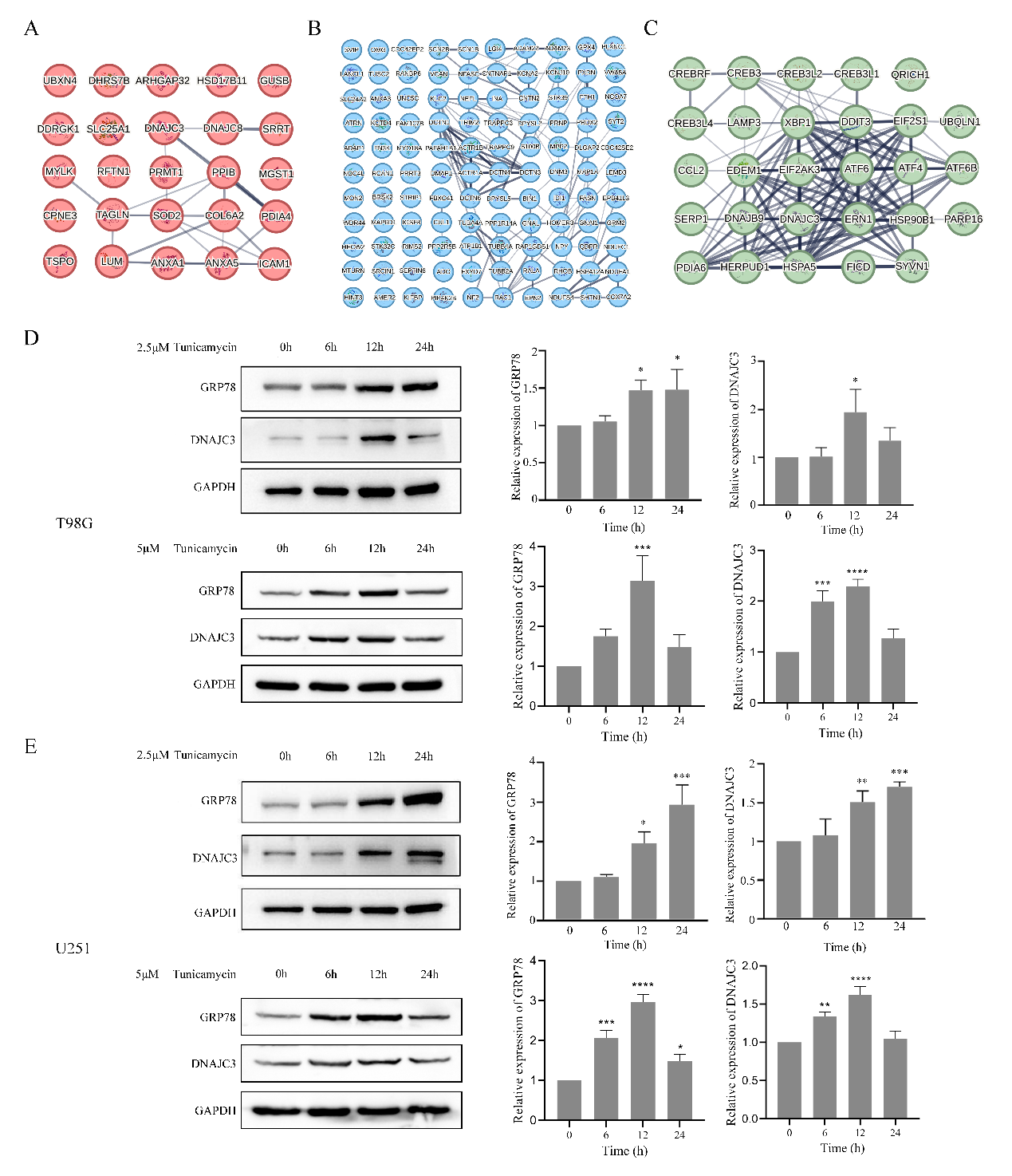
**

**Fig.S1 Screening for differentially expressed proteins.** (A) Significantly up-regulated differentially expressed proteins; (B) Significantly down-regulated differentially expressed proteins; (C) ERS-associated proteins; (D-E) After treating T98G and U251 cells with 2.5μM or 5μM tunicamycin, the changes in the expression levels of GRP78 and DNAJC3 with the extension of the treatment time (n = 3, ANOVA, compared with 0h). The data are presented as mean ± SD. ( ***P<0.001, **P<0.01, *P<0.05)

**
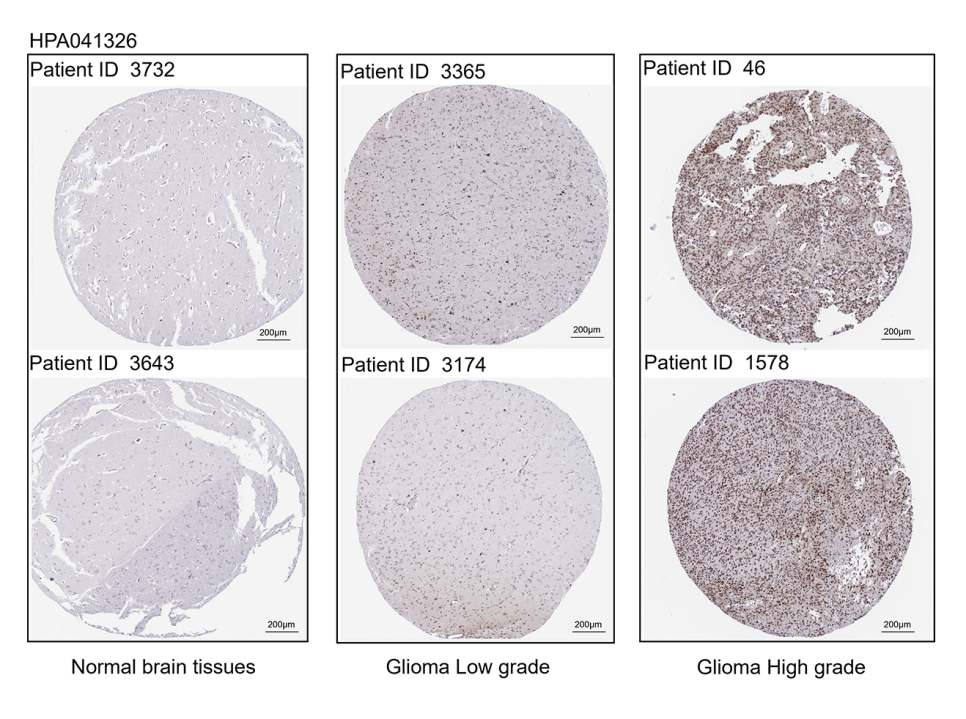
**

**Fig.S2 Representative IHC micrographs of DNAJC3 in brain samples, low grade glioma and high grade glioma from the HPA database.**


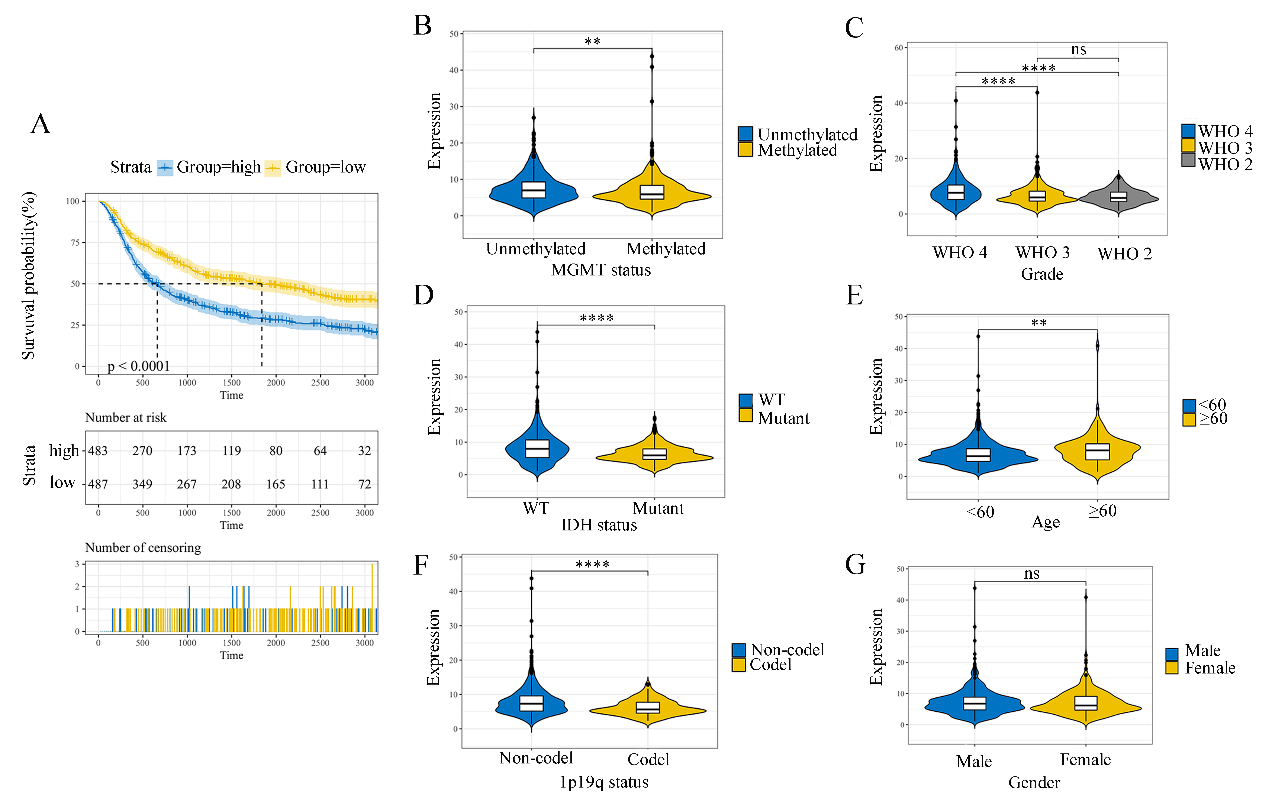


**Fig.S3 DNAJC3 was correlated with clinicopathological** **characteristics of glioma patients in the CGGA database.** (A) The relationship between high/low expression of DNAJC3 and the survival status of glioma patients (Log-rank test). (B-G) The relationship between high/low expression of DNAJC3 and the clinicopathological characteristics of glioma patients, including MGMT promoter methylation status (B), WHO grade (C), IDH mutation status (D), age (E), 1p19q co-deletion status (F), and gender (G) (Student's t-test/ ANOVA). (****P<0.0001; ***P<0.001; **P<0.01; *P<0.05; ns: no significance.)


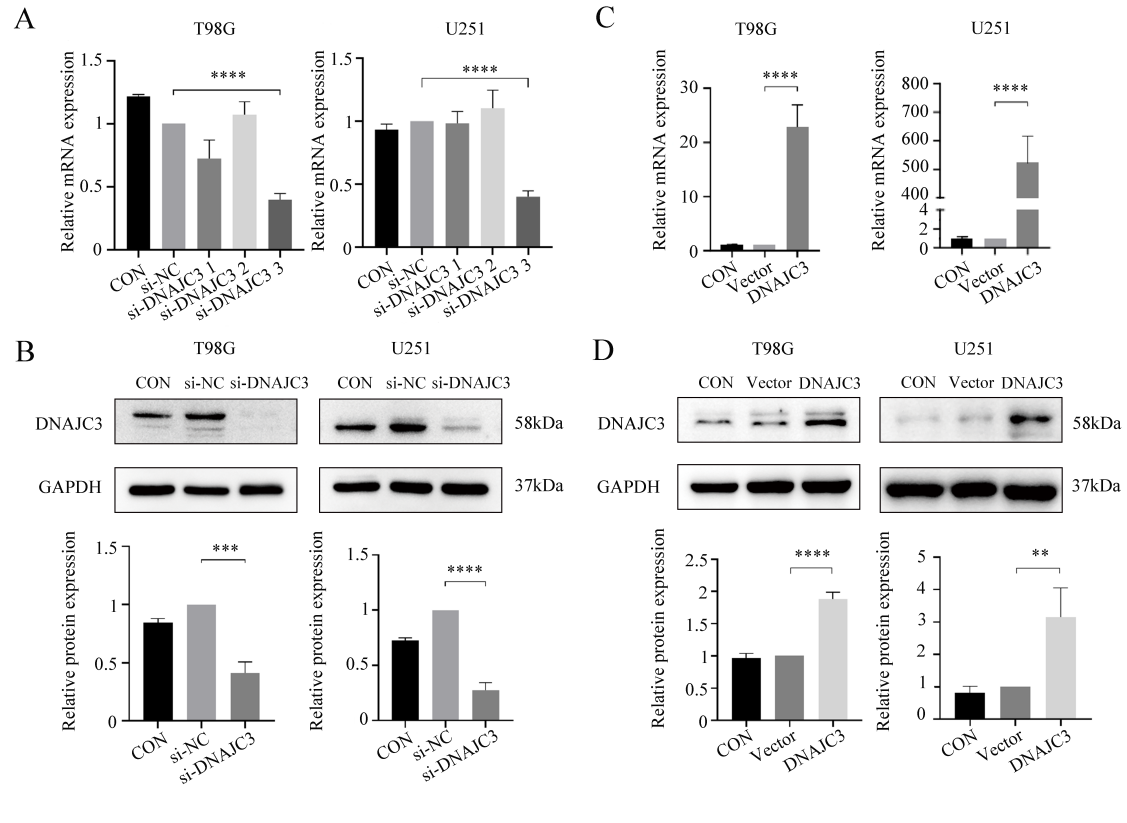


**Fig.S4 Validation of DNAJC3 knockdown/overexpression efficiency.** (A, C) QPCR was used to detect the knockdown (A) and overexpression (C) efficiency of the DNAJC3 gene (n = 3, ANOVA, compared with si-NC/Vector); (B, D) WB was used to detect the knockdown (B) and overexpression (D) efficiency of the DNAJC3 protein (n = 3, ANOVA, compared with si-NC/Vector). The data are presented as mean ± SD. ( ****P<0.0001; ***P<0.001; **P<0.01; CON: control group; si-NC: Negative control of the knockdown group; si-DNAJC3: DNAJC3 Knockdown group; Vector: Empty vector control of the overexpression group; DNAJC3: DNAJC3 overexpression group.)
